# Event and Comment

**Published:** 1911-06-15

**Authors:** 


### DENTAL REGISTER.

**Vol. LXV. June 15, 1911. No. 6**

### EVENT AND COMMENT.

EXAGGERATED CLAIMS FOR PROPRIETARY REMEDIES. It is well known that the most unscrupulous advertisers of patent medicines make exaggerated and unjustifiable statements as to the merits and universal application of their remedies and all intelligent people discount their statements accordingly. There seems to be at the present time a tendency on the part of the dental profession to take up with a class of remedies that undoubtedly have merit and a useful place in our work, but which are being forced into the notice of the profession through commercial sources too much after the methods of the unprofessional All sorts of root canal remedies, pyorrhea cures, and local anesthetics have been forced upon us almost to our disgust and certainly to the discrediting of our professional intelligence. We are now passing through a period of exploited remedies to be used for the prevention of dental caries, and some extremely exaggerated claims are being made in behalf of the many proprietary and secret preparations that the profession and oral hygienists are asked to endorse. We are not inclined to be too critical of this particular class of remedies, as they are in the main harmless and most people need to be stimulated to better care of the teeth. The worst that can be expected from them is that the consumers will find out sooner or later that the wonderful claims made for these remedies are not realized It seems to us that people, and especially an educated profession, ought to be able and willing to investigate impartially and conclusively any remedy which has sufficient merit to make its commercial promotion practicable, and that we should look with suspicion on all remedies which make extreme or even unaccustomed claims. All remedies and especially this class ought to be investigated promptly by competent authorities and their promoters should be made to keep their claims within the bounds of facts and reasonable inferences, and the public should be made acquainted with their true merits. The American Medical Association is doing this kind of work with some opposition, of course, from interested promoters, but with real satisfaction to the profession. It is also an effective means o keeping the profession from the habit of perfunctory prescribing, and of carelessness in diagnosis. In our profession there is a lack of discriminating use of the various drugs used in root canal treatment that promises a tremendous lot of future trouble, and in too many cases more immediate disaster obtains. If we could only get to the place where we were as willingly to know thoroughly our therapeutics as we seem anxious to develop our technical skill we should not only avoid many serious blunders, but we would to our own satisfaction settle our professional standing.

A CALL FOR A NEW DEFENSE ORGANIZATION. In another place we print the call for a convention to organize a new Protective Association. As to the wisdom of this move we are somewhat in doubt, because of the feeling existing between the persons most interested. It is usually the case that any movement initiated during a period of bitter controversy is likely to have a somewhat strenuous if not a disastrous outcome. We do not know just what the men who are calling this convention have in mind to propose; but we would suggest that whatever is done that personal animosities shall be set aside and that the general good of the profession may be kept constantly in mind. We have hoped that the Taggart suits might have been settled by this time. So that we should know what our rights and obligations might be, and every one then would be able to act intelligently and professionally. The great problem in this case seems to be to harmonize our professional ideals and our commercial obligations. If Taggart were not a professional man it would not take us long to adjust our relations: but he has not been able or willing to cut loose from his professional relations, and the profession seems loth to treat him as a business man. Can we not look upon this phenomenon as a kind of dental "Jekyll and Hyde" tragedy, and apply some kind of "Keeley Cure" treatment that will be less drastic than the impending and antagonistic contest which can only adjust the difficulty after a long and tiresome struggle which will bring little good to any one.

